# Secondary ovarian germ cell tumor following Wilms tumor after eight years post-treatment: A rare case report

**DOI:** 10.1016/j.ijscr.2025.111107

**Published:** 2025-03-03

**Authors:** Shahla Ansari-Damavandi, Yeganeh Pakbaz, Elham Zarei, Shiva Shadani

**Affiliations:** a Department of Pediatric Hematology and Oncology, School of Medicine, Iran University of Medical Sciences, Tehran, Iran; b Breast Health & Cancer Research Center, School of Medicine, Iran University of Medical Sciences, Tehran, Iran; c Department of Radiology, School of Medicine, Iran University of Medical Sciences, Tehran, Iran

**Keywords:** Wilms tumor, Secondary neoplasms, Ovarian germ cell tumor, Pediatric neoplasms, Case report

## Abstract

**Introduction:**

Wilms tumor (WT), or nephroblastoma, is a rare pediatric renal malignancy with generally favorable outcomes after multimodal treatment. However, survivors face a risk of developing secondary malignant neoplasms (SMNs), though these are relatively uncommon. This report details a rare case of a secondary germ cell tumor of the ovary occurring in an 11-year-old girl, eight years post-treatment for stage III WT. The case underscores the importance of extended surveillance in WT survivors for detecting late-onset SMNs.

**Presentation of case:**

An 11-year-old girl, previously treated for stage III WT with nephrectomy, chemotherapy, and abdominal radiotherapy, presented with abdominal distension. Physical examination revealed a palpable abdominal mass. Imaging studies, including ultrasound and CT scan, identified a large heterogeneous ovarian mass with cystic and necrotic areas, suggestive of malignancy. The mass was surgically excised, and histopathology confirmed a stage III germ cell tumor of the ovary. Due to metastasis to the omentum and lungs, additional surgical interventions, including supracervical hysterectomy and bilateral salpingo-oophorectomy, were performed.

**Discussion:**

This case highlights the potential for late-onset SMNs in WT survivors, particularly in patients with a family history of malignancies, emphasizing the need for long-term follow-up beyond the typical five-year period.

**Conclusion:**

The delayed appearance of secondary malignancies points to the necessity of multidisciplinary management and vigilant, prolonged surveillance to optimize outcomes for WT survivors.

## Highlights

• The patient's initial abdominal mass was later identified as a stage III germ cell tumor.
• The case emphasizes the need for follow-up beyond the conventional 5-year period.
• Managing secondary malignancies needs a multidisciplinary approach in WT survivors.
• It highlights the importance of continuous monitoring in pediatric oncology cases.

## 1 Introduction

Wilms tumor (WT), also known as nephroblastoma, is a rare pediatric malignancy characterized by an embryonal kidney mass. It affects approximately one in 10,000 children under the age of 15 in North America and Europe [1]. WT presents a significant clinical challenge due to its potential for aggressive behavior and metastasis [2]. The primary treatment typically involves a combination of surgical intervention, chemotherapy, and radiation therapy, tailored according to the disease stage at diagnosis [2,3].

Despite significant advancements in radiation therapy and chemotherapy during the 20th century, which have markedly improved survival rates from 10 % to 90 %, WT survivors still confront an ongoing risk of developing secondary malignant neoplasms (SMNs), a serious long-term complication [4]. The incidence of SMNs in WT survivors, though relatively rare, remains a concern, particularly among children with “favorable” histology and early diagnosis, who generally have a promising prognosis [5]. Recent studies have reported an increased prevalence of SMNs in WT survivors compared to the general population in Britain, Scandinavia, and North America [6,7].

In this case report, we present an unusual case of an 11-year-old girl who developed a secondary germ cell tumor of the ovary eight years after receiving treatment for stage III WT. This manuscript was prepared following the SCARE guidelines [8].

## 2 Case report

An 11-year-old girl was initially diagnosed with stage III WT at eight months of age. She underwent a right radical nephrectomy, chemotherapy, and whole abdomen radiation therapy. Eight years later, during a routine follow-up, the patient was referred for evaluation due to abdominal distension. Physical examinations revealed a palpable mass in the midline of the abdomen. An ultrasound demonstrated a large, heterogeneous cystic-solid mass measuring 75 × 130 mm, located in the supraumbilical region extending behind the uterus and bladder, with vascularity in solid areas. No abdominal lymphadenopathy was detected. Serological tests for alpha-fetoprotein (AFP) and beta-human chorionic gonadotropin (β-hCG) were normal, as were other serum parameters.

**Fig. 1:**
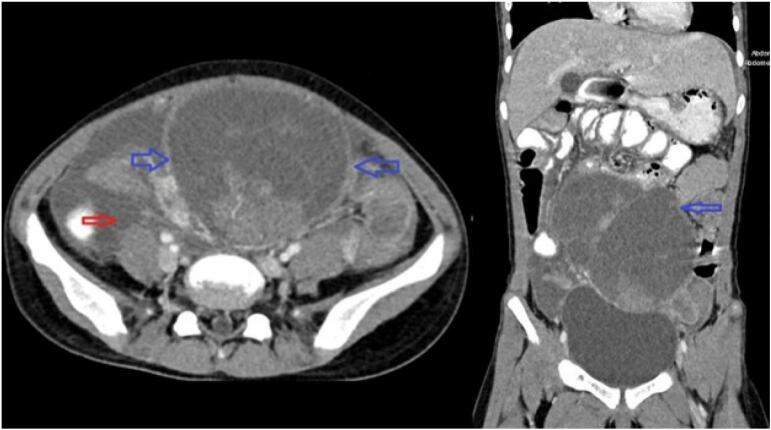
Axial and coronal views of a contrast-enhanced CT scan (IV and oral contrast) demonstrating a well-defined solid-cystic mass in the midline of the lower abdominal cavity, situated superior to the urinary bladder (blue arrows). The axial view also reveals the presence of ascites (red arrow). (For interpretation of the references to colour in this figure legend, the reader is referred to the web version of this article.)

**Fig. 2:**
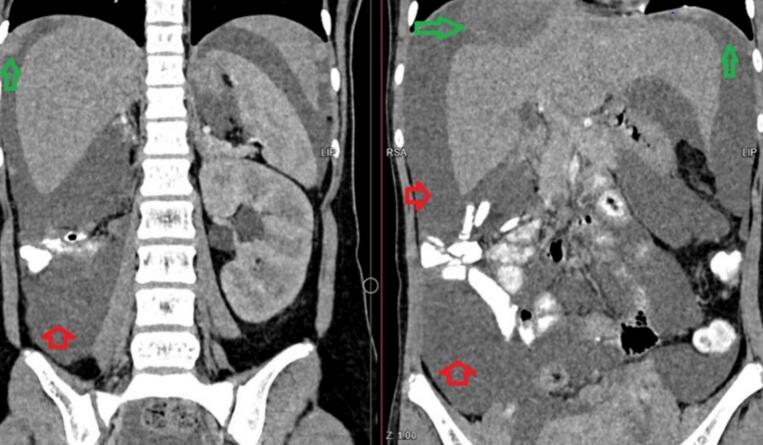
Coronal view of a contrast-enhanced CT scan (IV and oral contrast) illustrating ascites (red arrows) and peritoneal metastases (green arrows). (For interpretation of the references to colour in this figure legend, the reader is referred to the web version of this article.)

A computed tomography (CT) scan identified a large, well-defined, heterogeneously enhancing solid mass originating from the right ovary featuring several cystic and necrotic regions and associated with ascites (Fig. 1). It also showed evidence of peritoneal metastasis (Fig. 2). The tumor was surgically removed, and histopathological examination confirmed that it was a juvenile granulosa cell tumor of the ovary. It was also identified as a stage III ovarian germ cell tumor, a secondary malignancy arising eight years post-WT diagnosis.

Family history revealed breast cancer in the patient's mother and grandmother, and brain tumors in her brother, leading to death. Unfortunately, the patient did not attend follow-up appointments. A year later, she underwent a supracervical hysterectomy, left salpingo-oophorectomy, right salpingectomy, and chemotherapy due to metastasis of the germ cell tumor to the right adnexa, omentum, and lungs.

## 3 Discussion

WT, also known as nephroblastoma, is the most common renal cancer in children, the predominant abdominal cancer type, and the fourth most prevalent pediatric cancer overall. WT typically occurs in children under the age of five [9,10]. This case highlights the management and evaluation of a pediatric WT case with a rare secondary malignancy.

Paulino et al. mentioned that secondary malignancies are uncommon in WT patients, with only 1 % developing brain metastases [11]. Similarly, Shamsiah et al. reported that most extracranial pediatric solid tumors, including WT, rarely metastasize to the brain. Their case involved a stage IV WT with lung metastases evaluated five months post-treatment [12]. In contrast, our patient had stage III WT with secondary abdominal involvement.

Another case reported by Arion et al. was a seven-year-old girl with secondary vaginal involvement following a stage IV WT diagnosis with favorable histology. She was treated with radiation, chemotherapy, and surgery but presented with vaginal bleeding ten months after her last treatment [13]. Our case differs from these reports in terms of diagnosis timing and metastasis site.

There's growing evidence that some survivors of WT, particularly those with genetic predisposition syndromes, have a higher risk of developing secondary cancers. Genetic testing should be considered in these cases to better understand the hereditary factors and help guide surveillance strategies [14].

The current study confirms the significant risk of SMNs in patients treated for WT. Despite evidence suggesting that reduced use of radiotherapy can lower the risk of secondary solid tumors, the likelihood of severe secondary involvement seems to be increasing. Notably, the highest risk of SMNs is observed within the initial five years following WT diagnosis. Around 95 % of recurrences happen within the first two years post-diagnosis. Given the rarity of late recurrences, there is currently no established guideline for monitoring beyond five years post-diagnosis. Therefore, routine long-term surveillance for recurrence beyond this period is considered unnecessary [15,16]. However, in our case, secondary involvement occurred eight years post-diagnosis. This extended timeline for secondary malignancy emergence underscores the importance of long-term monitoring and follow-up for WT survivors to detect and manage late-onset complications effectively.

## 4 Conclusion

In conclusion, this case report adds to the limited literature on secondary malignancies following WT treatment, describing a rare and late occurrence of stage III WT with abdominal metastasis eight years after initial therapy. The development of SMNs in WT survivors, although rare, poses significant diagnostic and management challenges. Physicians should always be on the lookout for WT as a possible secondary malignancy in pediatric patients presenting with abdominal, uterine, or ovarian tumors, emphasizing the need for longer terms of follow-up and surveillance in WT survivors. Multidisciplinary collaboration between clinicians, radiologists, and pathologists is essential for accurate diagnosis and optimal management of secondary malignancies in WT survivors. This approach will help ensure favorable patient outcomes and improve the quality of life for survivors of this challenging pediatric malignancy. The extended timeline for secondary malignancy emergence in this case underscores the necessity of considering late-onset complications in the follow-up protocols for WT survivors. Early detection and intervention remain key to managing these secondary malignancies effectively, highlighting the importance of comprehensive and continuous monitoring beyond the conventional five-year surveillance period.

## Consent

Written informed consent was obtained from the patient's parents/legal guardian for publication and any accompanying images. A copy of the written consent is available for review by the Editor-in-Chief of this journal on request.

## Ethical approval

This case report was exempt from ethical approval by the Ethics Committee in Research at Iran University of Medical Sciences, as per institutional guidelines for case reports.

## Guarantor

Dr. Shahla Ansari-Damavandi and Dr. Shiva Shadani

## Author contributions

Shahla Ansari-Damavandi: Conceptualization; Writing - Review & Editing.

Yeganeh Pakbaz: Writing - Original Draft; Writing - Review & Editing; Software.

Elham Zarei: Investigation; Writing - Original Draft.

Shiva Shadani: Writing - Original Draft; Writing - Review & Editing; Conceptualization; Supervision.

## Sources of funding

None.

## Data sharing

Data sharing is not applicable to this article as no new data were created or analyzed in this study.

## Declaration of competing interest

The authors declare no conflict of interest.

## References

[1] Stiller C.A., Parkin D.M. (1990). International variations in the incidence of childhood renal tumours. Br. J. Cancer.

[2] Spreafico F., Fernandez C.V., Brok J., Nakata K., Vujanic G., Geller J.I. (2021). Wilms tumour. Nat Rev Dis Primers..

[3] Nelson M.V., van den Heuvel-Eibrink M.M., Graf N., Dome J.S. (2021). New approaches to risk stratification for Wilms tumor. Curr. Opin. Pediatr..

[4] D’Angio G.J. (1985). Oncology seen through the prism of Wilms tumor. Med. Pediatr. Oncol..

[5] Mardanpour K., Rahbar M., Mardanpour S., Mardanpour N., Rezaei M. (2020). CD8+ T-cell lymphocytes infiltration predict clinical outcomes in Wilms’ tumor. Tumour Biol..

[6] Cunningham M.E., Klug T.D., Nuchtern J.G., Chintagumpala M.M., Venkatramani R., Lubega J., Naik-Mathuria B.J. (2020). Global disparities in Wilms tumor. J. Surg. Res..

[7] Gadd S., Huff V., Skol A.D., Renfro L.A., Fernandez C.V., Mullen E.A. (2022). Genetic changes associated with relapse in favorable histology Wilms tumor: a Children’s Oncology Group AREN03B2 study. Cell Rep Med..

[8] Sohrabi C., Mathew G., Maria N., Kerwan A., Franchi T., Agha R.A. (2023). The SCARE 2023 guideline: updating consensus surgical CAse REport (SCARE) guidelines. Int. J. Surg..

[9] Breslow N.E., Palmer N.F., Hill L.R., Buring J., D’Angio G.J. (1978). Wilms’ tumor: prognostic factors for patients without metastases at diagnosis: results of the National Wilms’ tumor study. Cancer.

[10] Liu Y., Nelson M.V., Bailey C., Zhang P., Zheng P., Dome J.S. (2021). Targeting the HIF-1α-IGFBP2 axis therapeutically reduces IGF1-AKT signaling and blocks the growth and metastasis of relapsed anaplastic Wilms tumor. Oncogene.

[11] Paulino A.C., Nguyen T.X., Barker J.L. (2003). Brain metastasis in children with sarcoma, neuroblastoma, and Wilms’ tumor. Int. J. Radiat. Oncol. Biol. Phys..

[12] Shamsiah A.H., Yaakup N.A. (2015). Brain metastasis in a Wilm’s tumor patient: a case report. Middle East Journal of Cancer..

[13] Arion K., Dufour S., Ramphal R., Villani A., Malkin D., Shlien A. (2023). Vaginal metastases of Wilms’ tumor in a pediatric patient: a rare case. J. Pediatr. Adolesc. Gynecol..

[14] Maciaszek J.L., Oak N., Nichols K.E. (2020). Recent advances in Wilms’ tumor predisposition. Hum. Mol. Genet..

[15] Travis L.B., Rabkin C.S., Brown L.M., Allan J.M., Alter B.P., Ambrosone C.B. (2006). Cancer survivorship--genetic susceptibility and second primary cancers: research strategies and recommendations. J. Natl. Cancer Inst..

[16] Malogolowkin M., Spreafico F., Dome J.S., van Tinteren H., Pritchard-Jones K., van den Heuvel-Eibrink M.M. (2013). Incidence and outcomes of patients with late recurrence of Wilms’ tumor. Pediatr. Blood Cancer.

